# Molecules Altering the Intracellular Thiol Content Modulate NF-kB and STAT-1/IRF-1 Signalling Pathways and IL-12 p40 and IL-27 p28 Production in Murine Macrophages

**DOI:** 10.1371/journal.pone.0057866

**Published:** 2013-03-11

**Authors:** Alessandra Fraternale, Rita Crinelli, Anna Casabianca, Maria Filomena Paoletti, Chiara Orlandi, Elisa Carloni, Michaël Smietana, Anna Teresa Palamara, Mauro Magnani

**Affiliations:** 1 Department of Biomolecular Sciences University of Urbino “Carlo Bo”, Urbino (PU), Italy; 2 Institut des Biomolécules Max Mousseron, UMR 5247 CNRS Université Montpellier 1, Université Montpellier 2, Montpellier, France; 3 Department of Public Health and Infectious Diseases, Pasteur Institute, Cenci-Bolognetti Foundation, “Sapienza” University of Rome, Rome, Italy; 4 San Raffaele Pisana Scientific Institute for Research, Hospitalization, and Health Care, Rome, Italy; National Jewish Health and University of Colorado School of Medicine, United States of America

## Abstract

**Background:**

The aim of this study was to investigate the molecular mechanisms involved in the production of Th1 cytokines, namely IL-12 and IL-27, when the intra-macrophage redox state was altered by different chemical entities such as GSH-C4, which is reduced glutathione carrying an aliphatic chain, or I-152, a pro-drug of N-acetyl-cysteine (NAC) and beta-mercaptoethylamine. We had already demonstrated that GSH-C4 and I-152 could shift the immune response towards Th1 in Ovalbumin-immunized mice as well as enhance Th1 response in HIV-1 Tat-immunized mice.

**Methodology/Principal Findings:**

By a new high performance liquid chromatography method, we found that 20 mM GSH-C4 provided a number of thiol species in the form of GSH, while 20 mM I-152 decreased GSH and increased the thiols in the form of NAC and I-152. Under these experimental conditions, GSH-C4 and I-152 enhanced and suppressed respectively the mRNA expression levels of IL-12 p40 induced by LPS/IFN-γ as assessed by Real-Time PCR. The protein production of IL-12 p40 was increased by GSH-C4 and decreased by I-152 as determined by Enzyme-linked immunosorbent assay. Western immunoblot and electrophoretic mobility shift assays revealed that Nuclear Factor -kB (NF-kB) activation was inhibited by I-152 and prolonged by GSH-C4. Twenty mM I-152 stimulated IL-27 p28 gene expression and sustained Signal Transducer and Activator of Transcription (STAT)-mediated interferon regulator factor 1 (IRF-1) de novo synthesis. By contrast, 20 mM GSH-C4 did not exert any effect on IL-27 p28 gene expression.

**Conclusions and Significance:**

an increase in the intra-macrophage redox state by GSH-C4 and I-152 enhances Th1 cytokine production although the chemical structure and the intra-cellular metabolism influence differently signalling pathways involved in IL-27 or IL-12 production. GSH-C4 and I-152 may be used as Th1 immunomodulators in some pathologies and in ageing where GSH depletion may contribute to the Th1/Th2 imbalance, and in new immunization strategies.

## Introduction

CD4+T helper cells differentiate into specialized types of effector cells: type 1 (Th1), type 2 (Th2) or the recently identified IL-17 producing (Th17) helper T cells, which promote different aspects of the immune response. An appropriate Th1/Th2 balance is critical for an effective and non pathological immune function. Many factors influence the decision as to whether a Th1 or Th2 cytokine pattern predominates in a given response and, among these, a strong link between reduced glutathione (GSH) and immune function has been established in various studies. The role of cytokines and the critical switch between Th1 and Th2 is important in several diseases such as cancer, allergic disorders and AIDS; all these pathologies are characterized by GSH depletion in antigen-presenting-cells (APC) and by Th1 failure. In vitro and in vivo studies demonstrated that GSH depletion in APC correlates with defective antigen processing and reduced Th1-associated cytokine production [1]–[7]. In murine macrophages, GSH depletion caused reduction of interleukin-12 (IL-12) secretion and led to a switch from the typical Th1 cytokine profile towards Th2 response patterns [6]. In human alveolar macrophages, GSH levels played a pivotal role in determining whether a Th1 or Th2 cytokine response would develop [7]. In human monocyte-derived DCs (MD-DCs), the molecules able to increase and decrease intracellular GSH content could augment and reduce lipopolysaccharide (LPS)-induced IL-27 respectively [8]. Thus, expression of both IL-12 and IL-27 seem to be profoundly influenced by the redox state of APC. Cytokines have emerged as key determinants in the developmental process of CD4+ T cells into Th1 or Th2 cells [9]. IL-12, a heterodimer of the p40 and p35 subunits, is an important immunoregulatory cytokine that is produced mainly by APC; it is a dominant factor in driving the development of Th1 cells leading to secretion of interferon γ, which stimulates the immune response to eradicate intracellular pathogens [10]. Molecules able to augment the intracellular GSH/GSSG ratio in APC were found to increase the expression of LPS-induced IL-12 mRNA and ameliorate “Th2” diseases by altering the Th1/Th2 balance [11]–[13]. IL-27, a novel IL-12 family cytokine, is a heterodimeric molecule composed of Epstein-Barr virus-induced gene 3 (EBI3), an IL-12 p40-related protein, and p28, an IL-12 p35-related polypeptide. IL-27 is produced early by activated APC in response to microbial infection. It is able to induce clonal proliferation of naïve CD4+ T cells and synergizes with IL-12 in IFN-γ production [14]. In detail, IL-27 activates STAT-1 and T-bet, two transcription factors that are important for Th1 polarization, and leads to up-regulation of the IL-12Rβ2 chain. In this way, IL-27 sensitizes the cells to Th1 enhancing cytokine IL-12. Meanwhile, it also suppresses the other helper cell types by suppressing Th2 transcription factor GATA-3 [15]. In our previous papers, we reported the capacity of two molecules, able to alter the intracellular redox state, to modulate the immune response towards Th1 type in Ova-sensitized mice and to potentiate the immune response of another antigen, i.e. Tat, which is in clinical trials for the development of anti-HIV/AIDS vaccine [16], [17]. The molecules investigated were a pro-drug of N-acetyl-cysteine (NAC) and beta-mercaptoethylamine (MEA), named I-152, and the N-butanoyl GSH derivative, GSH-C4, which had already been used successfully as antiviral [18]–[21]. Here, we wanted to investigate the molecular mechanisms by which GSH-C4 and I-152 could control the macrophage cytokine profile. In the current study, we demonstrate that 20 mM I-152 inhibited signal-induced Nuclear Factor -kB (NF-kB) nuclear translocation by blocking Inhibitor –kB-α (IkB-α) degradation. This effect could explain the observed reduction of IL-12 p40 expression in macrophages. At the same concentration, I-152 sustained STAT-1-dependent IRF-1 activation and increased IL-27 p28 gene transcription. By contrast, GSH-C4 prolonged LPS/IFN-γ-induced NF-kB activation and enhanced IL-12 p40 production, while it had no effect on IL-27 production.

## Results

### GSH-C4 and I-152 Affect Intra-macrophage Thiol Content Differently

**Figure 1:**
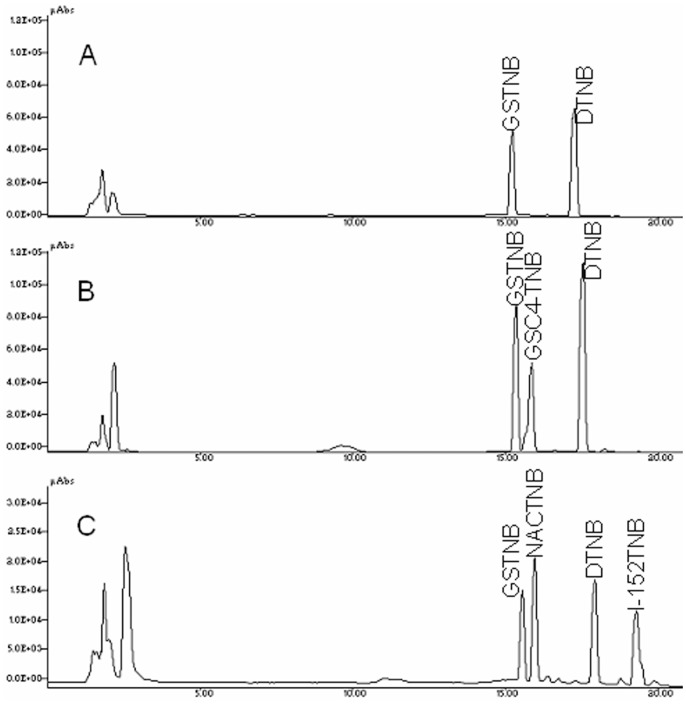
Separation of DTNB and R-TNB by reversed-phase HPLC. (A) extracts of untreated murine peritoneal macropahges; (B) extracts of murine peritoneal macrophages treated with 20 mM GSH-C4 for 2 hours; (C) extracts of macrophages treated with 20 mM I-152 for 2 hours. Murine macrophages were obtained and processed as described in the Materials and Methods section. Experimental details and chromatographic conditions are described in the Materials and Methods section.

The thiol content was determined both in murine peritoneal macrophages and in RAW 264.7 cells after treatment with various concentrations of either GSH-C4 or I-152. The cells were treated with increasing concentrations of the molecules for 2 h, and intracellular thiol content was quantified by HPLC as described in the Materials and Methods section. The new method developed is based on the reaction of Ellman’s reagent (5,5′-dithiobis-(2-nitrobenzoic acid) or DTNB) with the thiol group of the molecule used (R-SH); the thiols react with DTNB cleaving the disulfide bond resulting in thionitrobenzoate (TNB^-^) and mixed disulfide (R-TNB). The TNB^-^ ionizes to the TNB^2−^ in water and can be quantified in a spectrophotometer by measuring the absorbance at 412 nm. The R-TNB can be separated and quantified by HPLC by the method described. This method permits the distinction of GSH from GSH-C4 in the cells treated with GSH-C4, and GSH from other thiol species such as I-152 and NAC present in the cells treated with I-152. Fig. 1 shows a representative chromatographic profile of the extracts obtained from peritoneal macrophages untreated (Control) (A) or treated with either 20 mM GSH-C4 (B) or 20 mM I-152 (C).

**Figure 2:**
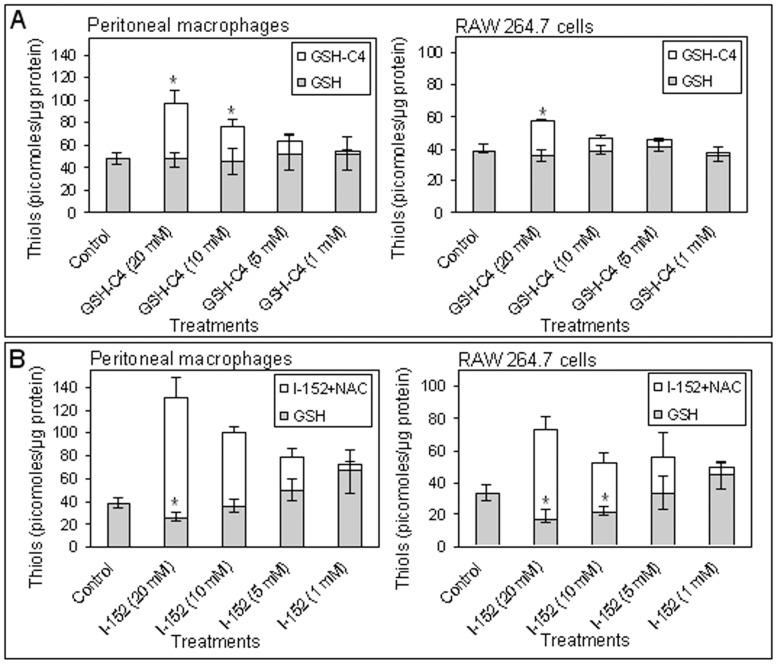
Quantification of thiol species by HPLC. The analysis was performed in murine peritoneal macrophages (left) and RAW 264.7 cells (right) treated with different concentrations of GSH-C4 (A) and I-152 (B). The peritoneal macrophages were obtained and processed as described in the Materials and Methods section. Values represent the mean±S.D. of 3 experiments. *p<0.05 vs. Control.

Incubation with GSH-C4 resulted in a dose-dependent increase in intracellular GSH, represented by the sum of intracellular GSH plus GSH-C4, in murine peritoneal macrophages (p<0.05 in 20 and 10 mM GSH-C4-treated) (Fig. 2A, left). An increase in total GSH content over the basal level was also observed in RAW 264.7 cells, although the overall levels of GSH were more modestly affected compared to murine macrophages (p<0.05 in 20 mM GSH-C4 treated) (Fig. 2A, right). In both models, only with slight differences, pre-treatment with I-152 altered thiol content in a quantitative and qualitative manner depending on the amount of molecule used (Fig 2B). High concentrations of I-152 (10 and 20 mM) decreased the GSH levels (p<0.05 in 10 and 20 mM I-152-treated RAW 264.7 cells and in 20 mM I-152-treated peritoneal macrophages) while, as expected, high concentrations of thiols, consisting of NAC plus I-152, were found. Lower concentrations of I-152 (5 and 1 mM) increased both the thiol content and GSH levels when compared to the control (Fig. 2B left). Differences in GSH levels were almost significant in 5 and 1 mM I-152-treated peritoneal macrophages (p = 0.076 and p = 0.088 respectively).

### Modification of Intracellular Thiol Content by GSH-C4 and I-152 Affects IL-12 p40 and IL-27 p28 Production in Murine Macrophages

Based on the results of Fig. 2, which show that the most significant differences in terms of thiol content were obtained by treatment with the highest concentration tested (i.e. 20 mM), IL-12 and IL-27 production was examined in peritoneal macrophages treated with 20 mM of either GSH-C4 or I-152. IL-12 is a heterodimeric protein of 70 kDa, of which the p35 subunit is constitutively expressed at lower level by a variety of cell types, whereas the p40 subunit is only produced by the cells to make biologically active IL-12 [22].

**Figure 3:**
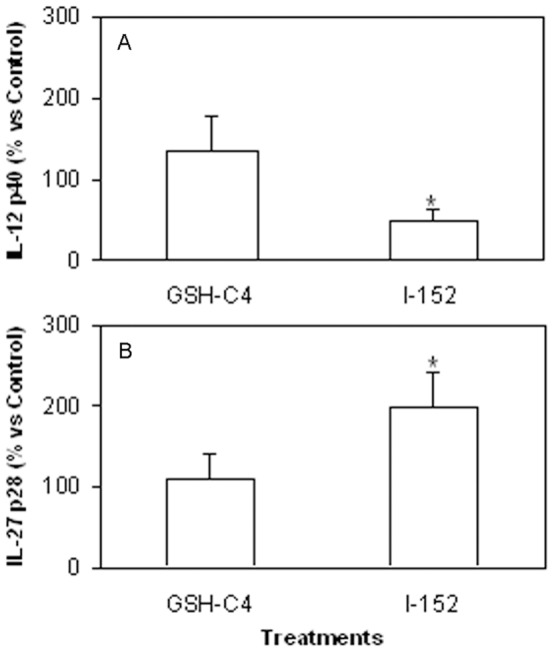
IL-12 p40 and IL-27 p28 induction in murine peritoneal macrophages. The peritoneal macrophages were obtained as described in the Materials and Methods section. They were left untreated or treated with either 20 mM GSH-C4 or 20 mM I-152 for 2 hours and further stimulated with LPS and IFN-γ for 24 hours. The IL-12 p40 (A) and IL-27 p28 (B) levels secreted in the culture supernatants were measured by ELISA. Results are expressed as the percentage respect to untreated stimulated macrophages (Control) and are the mean±S.D. of al least 5 different experiments. *p<0.05 vs. Control.

Peritoneal macrophages were pre-treated with GSH-C4 or I-152 for 2 h, followed by stimulation with LPS plus IFN-γ for 24 h. At the end of the incubation time, the secretion of the cytokine in the culture medium was evaluated by ELISA. It was observed that IL-12 p40 was more abundant in the culture medium of GSH-C4-treated macrophages than in untreated cells, following stimulation (123±37 picograms/µg protein in GSH-C4-treated cells and 92±30 picograms/µg protein in untreated cells). Differences were almost significant (p = 0.083). On the contrary, the levels of the p40 subunit were significantly reduced by I-152 (39±17 picograms/µg protein) (p<0.05) (Fig. 3A).

IL-27 synergizes with IL-12 in IFN-γ production from naïve CD4+ T cells and plays an important role in host defence against infection; defects in the IL-27 receptor causes increased susceptibility to different intracellular pathogens and it has been reported that Th1 responses can be controlled by intra-cellular glutathione redox status in dendritic cells (DCs) through IL-27 production [8], [23], [24]. Thus, in the macrophages treated as above, the IL-27 p28 levels were measured as well and found to have increased more than two times compared to control by I-152 (p<0.05) (30.4±5.3 picograms/µg protein in I-152-treated cells and 15.7±1.7 picograms/µg protein in untreated cells), while GSH-C4 had no effects (Fig. 3B).

**Figure 4:**
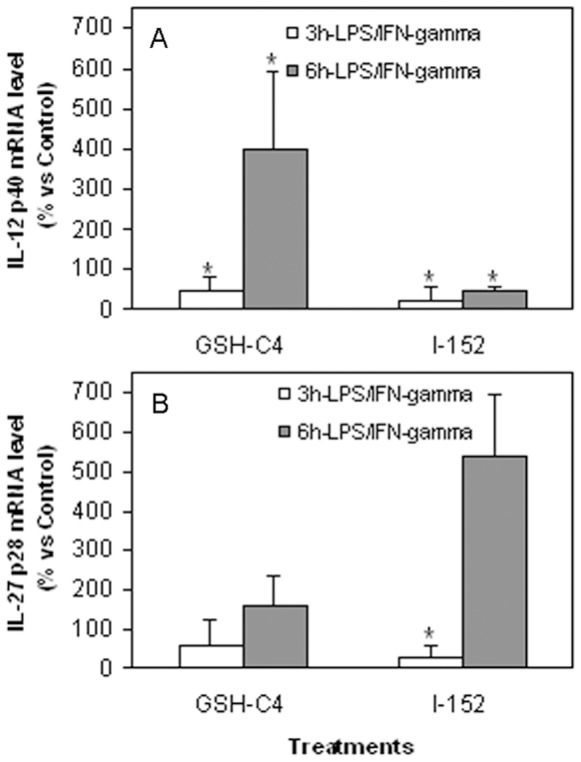
Expression of IL-12 p40 and IL-27 p28 mRNA in murine peritoneal macrophages. The peritoneal macrophages were obtained as described in the Materials and Methods section. They were left untreated or treated with either 20 mM GSH-C4 or 20 mM I-152 for 2 hours and further stimulated with LPS and IFN-γ for 3 and 6 hours as indicated in the figure. Total RNA extraction and reverse transcription as well as IL-12 p40 and IL-27 p28 mRNA quantification were performed as described in the Materials and Methods section. The results are the mean±S.D. of 4 values. *p<0.05 vs. Control.

The fluctuations in the levels of IL-12 p40 protein in response to GSH-C4 and I-152, respectively, were also reflected at the mRNA level; mRNA content was evaluated in macrophages stimulated with LPS plus IFN-γ for 3 h and 6 h. In Fig. 4A it can be observed that GSH-C4 inhibited IL-12 p40 mRNA expression at 3 h, but at 6 h mRNA levels were significantly up-regulated (almost 4-fold) compared to control (p<0.05). By contrast, p40 mRNA levels were significantly lower in cells treated with I-152 both at 3 h and 6 h (p<0.05). Different effects were exerted by I-152 on IL-27 p28 mRNA; in fact, at 3 h after stimulus p28 mRNA expression appeared lower compared to control, however, at 6 h it was about five times higher (p = 0.0571); treatment with GSH-C4 did not produce effects significantly different compared to control macrophages at both time points studied (Fig. 4B).

Peritoneal macrophages incubated with either 20 mM GSH-C4 or 20 mM I-152 were still metabolically active after 4 h, as assessed by MTS assay, and at 24 h about 80% of the macrophages treated with GSH-C4 and 90% of the macrophages treated with I-152 were still alive (not shown).

### GSH-C4 and I-152 Differently Affect Immune Signalling Pathways Involved in IL-12 p40 and IL-27 p28 Expression

**Figure 5:**
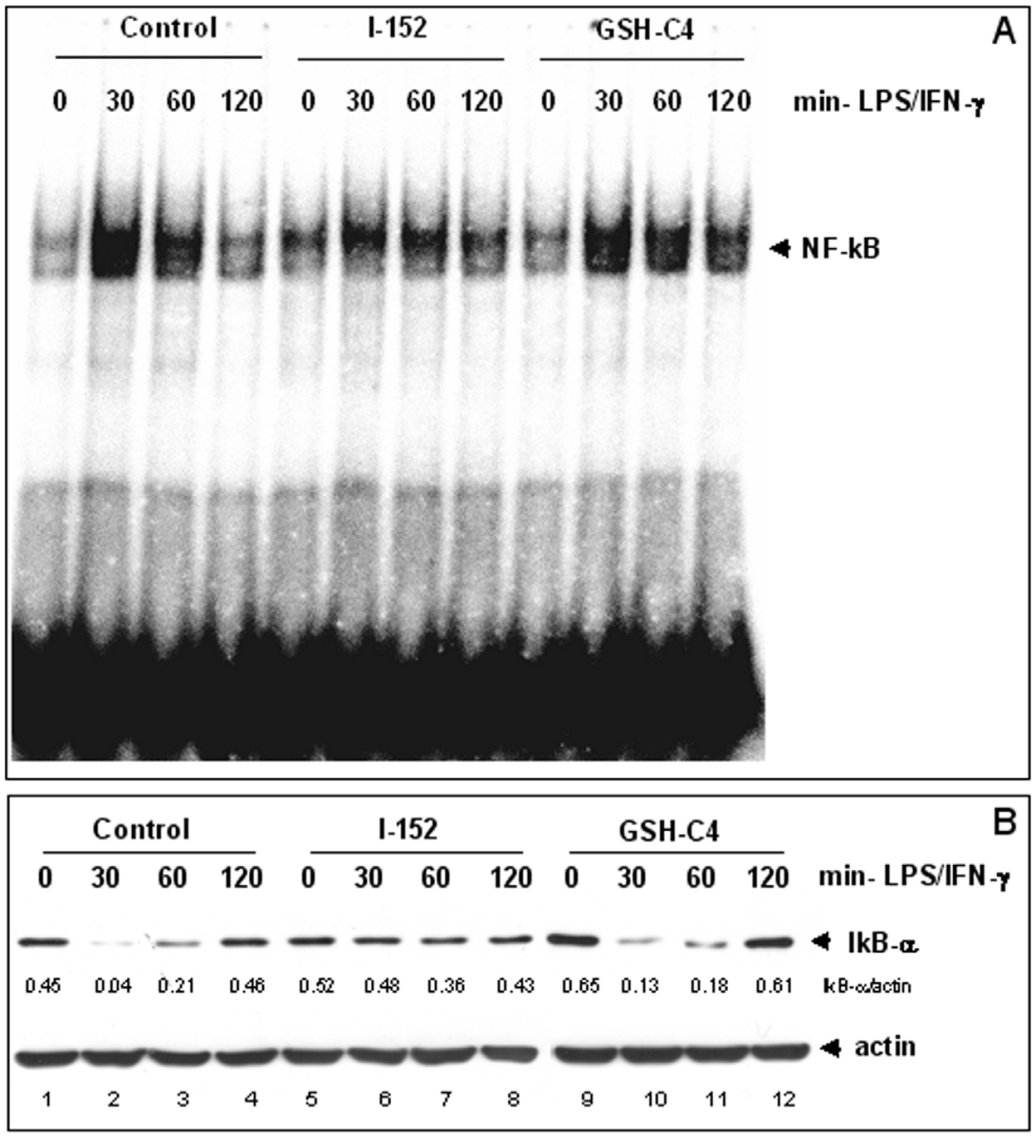
NF-kB activation in murine peritoneal macrophages. (A): NF-kB nuclear translocation and DNA binding activity was assessed by Electrophoretic Mobility Shift Assay (EMSA) of nuclear extracts obtained from macrophages untreated (Control) or pre-treated with either 20 mM GSH-C4 or 20 mM I-152 for 2 hours and then stimulated with LPS and IFN-γ after molecule removal, for different times as indicated. (B): IkB-α degradation and re-synthesis as monitored by Immunoblotting analysis of IkB-α protein levels in the cytosolic fraction of macrophages treated as above. Cytosolic proteins (15 µgs) were separated by SDS-PAGE onto 10% acrylamide gels, transferred to nitrocellulose membrane and immunoblotted with an anti-IkB-α antibody. Actin was stained as a loading control. IkB-α densitometric values, normalized to actin, are reported immediately below the IkB-α blot.

We next examined whether GSH-C4 and I-152 might interfere with the activation of transcription factors known to be involved in IL-12 p40 and IL-27 p28 expression in response to bacterial LPS and IFN-γ. Multiple regulatory elements are implicated in p40 regulation but the p50/p65 NF-kB rel proteins are known to dominantly regulate IL-12 p40 transcription [25]. Therefore, to evaluate NF-kB activation, murine peritoneal macrophages were either left untreated or treated with the single molecules for 2 h followed by stimulation with LPS plus IFN-γ for 30, 60 and 120 min. Nuclear extracts were then submitted to EMSA using a probe containing the –kB consensus sequence. NF-kB nuclear translocation was maximum in untreated cells (Control) as soon as 30 min after the stimulation (Figure 5A, lane 2) and decreased at the following time points to return to values similar to the basal level after 120 min (Fig. 5A, lane 4 vs. lane 1). A maximum peak of activation was also observed in GSH-C4-treated macrophages at 30 min (Fig. 5A, lane 10). However, the NF-kB signal appeared only slightly decreased at the following time points, especially at 120 min (Fig. 5A, lane 12), when NF-kB inactivation already occurred in the Control (Fig. 5A, lane 4). This suggests that the molecule presumably interferes with the dampening rather than with the activation of NF-kB signalling. By contrast, NF-kB DNA binding was slightly activated in cells treated with I-152 (Fig. 5A, lane 5 vs. lane 1) and its stimulus-induced nuclear translocation was reduced compared to Control (Fig. 5A, lanes 6–8 vs. lanes 2–4).

NF-kB is known to be controlled upstream by the cytoplasmic IkB-α, which sequesters NF-kB in the cytoplasm of quiescent cells [26], [27]. Upon macrophage activation, IkB-α is phosphorylated. Phosphorylation targets IkB-α for ubiquitination and degradation allowing NF-kB to translocate to the nucleus. Therefore, we examined whether the changes in the intracellular redox state induced by GSH-C4 and I-152 had a direct effect on the degradation of IkB-α (Fig. 5B). In Control cells, IkB-α degradation was maximum (Fig. 5B, lane 2) in coincidence with NF-kB nuclear translocation (i.e. 30 min, Fig. 5A, lane 2). At later times, the protein was re-synthesized and reappeared in the cytosol (Fig. 5B, lanes 3, 4), in agreement with the knowledge that NF-kB activation is transient and one of the mechanisms for termination involves the synthesis de novo of the IkB-α protein which can enter the nucleus, remove NF-kB from the DNA, and relocalize it to the cytosol [28]. Thus, in parallel with IkB-α re-synthesis, a decrease in the NF-kB DNA binding was observed in the nuclei (Fig. 5A, lanes 3, 4). Despite prolonged activation of NF-kB in GSH-C4-treated cells, both IkB-α degradation and re-synthesis occurred with the same kinetic of Control cells (Fig. 5B, lanes 9–12 vs. lanes 1–4), suggesting that other termination mechanisms could be affected. By contrast, I-152 treatment drastically reduced signal-induced IkB-α degradation (Fig. 5B, lanes 5–8 vs. lanes 1–4), thus explaining the observed inhibition of NF-kB nuclear translocation in I-152-treated cells. Thus, while I-152 inhibits, GSH-C4 seems to protract NF-kB activation, thus explaining the observed decrease and increase, respectively, of IL-12 p40 production in cells receiving the molecules compared to untreated macrophages.

**Figure 6:**
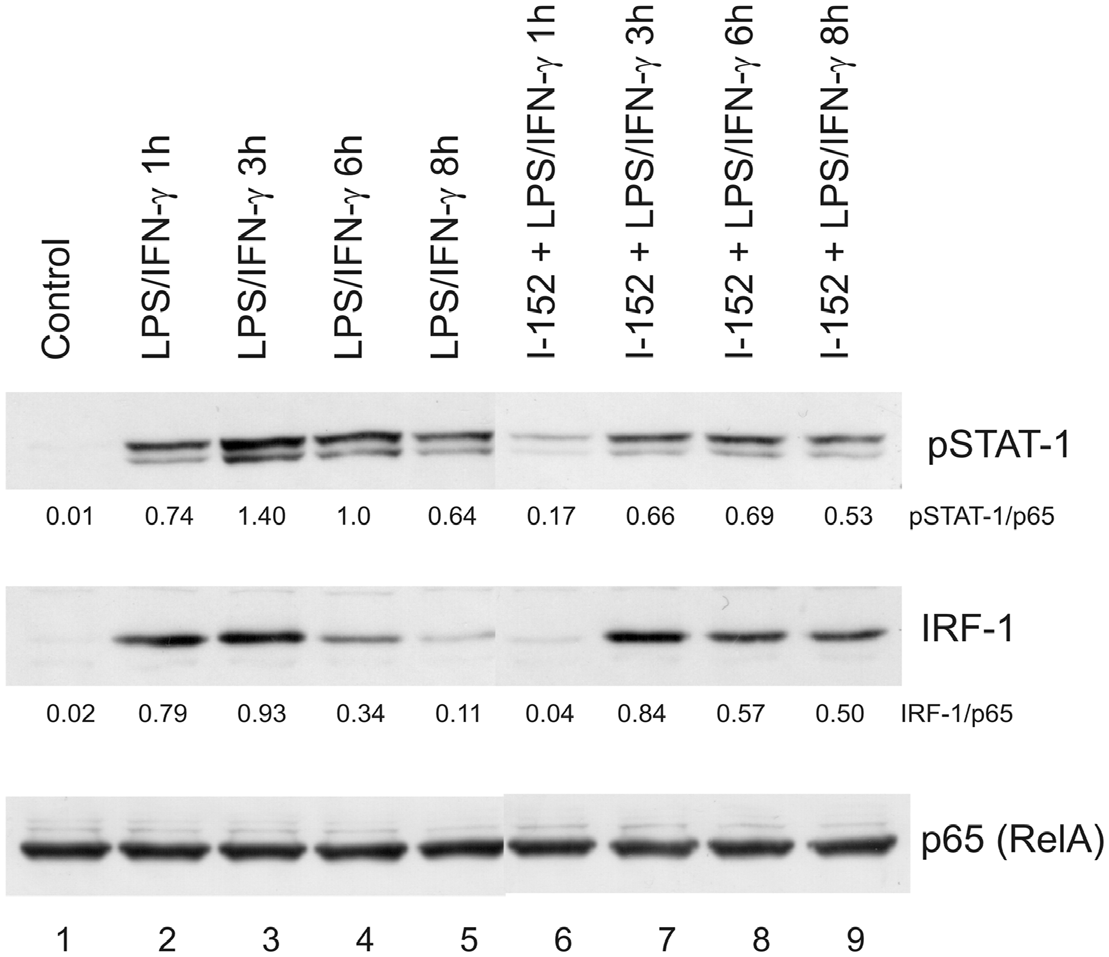
STAT-1 phosphorylation and IRF-1 levels in RAW 264.7 cells. Immunoblotting analysis of phospho Tyr701 STAT-1 and IRF-1 levels in cells untreated (lanes 1–5) or pre-treated with 20 mM I-152 for 2 hours (lanes 6–9) and then stimulated with LPS and IFN-γ after molecule removal, for different times as indicated (lanes 2–5 and 6–9). Whole protein extracts (20 µgs) were resolved onto 8% acrylamide gels, transferred to a nitrocellulose membrane and stained with anti IRF-1 and anti phospho Tyr701 STAT-1 specific antibodies (pSTAT-1). NF-kB p65 (RelA) was stained as a loading control. pSTAT-1 and IRF-1 densitometric values, normalized to p65, are reported immediately below the corresponding blot.

IRF-1 is the principal member of the IRF family of transcription factors activated by IFN-γ and is essential for many IFN-γ responses. It has been reported that IRF-1 is an essential transcription factor for p28 gene expression induced by IFN-γ in macrophages [29], [30]. We found that the treatment with 20 mM I-152 increased both p28 protein secretion and mRNA expression in macrophages stimulated by IFN-γ and LPS (Figs. 3B and 4B), so we investigated whether IRF-1 had a role in p28 gene expression induced by I-152. To this end, we performed EMSA using nuclear extracts obtained from murine macrophages or RAW 264.7 cells, treated with I-152 and stimulated with LPS/IFN-γ, and, as probe, a sequence of the p28 promoter harboring the known IRF-1 responsive elements [29]. Since in all cases the signal was weak (data not shown), we decided to follow IRF-1 activation by western immunoblotting. Indeed, it is known that type I and type II IFNs stimulate IRF-1 synthesis through the Janus kinase-STAT signalling which triggers the STAT1-mediated transcriptional activation of IRF-1 [31], [32]. In line with these findings, IRF-1 was undetectable in whole cell extracts derived from unstimulated RAW 264.7 cells (Control in Fig. 6, lane 1), while it appeared as soon as 1 h after stimulation with LPS/IFN-γ, in coincidence with STAT-1 phosphorylation on Tyr701 (Fig. 6 lane 2). It is well known that phosphorylation of STAT-1 at this residue induces its dimerization, nuclear translocation and DNA binding [33].

While in control cells, IRF-1 protein content was still high after 3 h of stimulation (Fig. 6, lane 3) to rapidly decline at later time points (i.e. 6 h and 8 h) (Fig. 6 lanes 4, 5), IRF-1 became detectable in I-152-treated cells at 3 h of LPS/IFN-γ stimulation in parallel with STAT-1 phosporylation (Fig. 6 lane 7) and its levels were unchanged within the next 3–5 h (i.e. 6 h and 8 h of stimulation) (Fig. 6 lanes 8, 9). As loading control, the blot was re-probed with an anti p65 antibody since, upon stimulation, p65 redistributes within intracellular compartments while the whole content does not change. On the whole, these findings suggest that I-152 on one hand delays STAT-1 activation and on the other sustains and prolongs the STAT-1/IRF-1 signalling. These results may justify the increased IL-27 p28 production observed in I-152-treated cells.

## Discussion

GSH levels in APC play a central role in determining whether Th1 or Th2 cytokine response patterns predominate in immune responses. GSH depletion leads to a shift away from the typical Th1 cytokine profile toward Th2 response patterns [4]–[7].

Th1 cytokine production is positively or negatively polarized by augmenting or depleting, respectively, the intracellular content of GSH in APC [4]–[8]. In previous papers we reported that administration of GSH-C4 and I-152 could shift the immune response towards Th1 both in mice immunized with Ova in which a prevalent Th2 response is developed and in mice immunized with HIV-1 Tat in which Th1 response is predominant [16], [17]. The pro-GSH molecules used in these studies were an aliphatic derivative of GSH, GSH-C4 [18] and a NAC/MEA conjugate compound, I-152 [20], [34] both designed to facilitate cell entry. In this paper, we investigate the molecular mechanisms whereby GSH-C4 and I-152 can influence the production of Th1 cytokines; in particular, we studied the LPS/IFN-γ-induced expression of both IL-12 p40 and IL-27 p28 in macrophages pre-treated with the molecules. The results obtained show that the two chemical entities exert different effects on the production of the above cytokines, probably due to their different intracellular metabolism. The developed HPLC method used in this paper allowed the distinction of the different thiol species generated intracellularly by GSH-C4 and I-152, while the spectrophotometric methods previously used [16] had provided total thiol content [35], [36]. This is a very simple method allowing an accurate study of the metabolism of the molecules of interest containing thiol groups. GSH-C4 provided a number of thiol species in the form of GSH, while high concentrations of I-152 reduced the intracellular GSH content and provided a high concentration of NAC and I-152. Although both molecules increased thiol content about 2–3 fold over the basal level, they displayed a very different effect on immune signalling pathway responsible for IL 12 p40 and IL-27 p28 expression, i.e. the NF-kB and the STAT-1/IRF-1 signalling pathways, suggesting that several investigations based on the sole determination of intracellular thiols are not adequate to understand the role of the cell redox state on Th1/Th2 polarization. It is known that the canonical signalling transduction pathway of NF-kB activation, which involves IkB phosphorylation and degradation, is sensitive to oxidant/antioxidant homeostasis, especially to the thiol/disulphide balance [37]. Data presented demonstrate that the thiol increase in the form of GSH, induced by GSH-C4 treatment, does not alter NF-kB activation in response to LPS/IFN-γ; in fact, IkB-α degradation and NF-kB nuclear translocation occurred to a similar extent and with the same kinetic as in Control cells. GSH-C4 rather interferes with the regulatory mechanism(s) that determine NF-kB inactivation. In particular, prolonged NF-kB activation was observed in GSH-C4-treated cells compared to stimulated control cells, an observation which could explain the accumulation of IL-12 p40 mRNA and protein in GSH-C4-treated cells. The best known mechanism for termination of NF-kB response involves re-synthesis of IkB proteins induced by activated NF-kB. However, IkB-α protein was re-synthesized in GSH-C4-treated cells to the same extent and with the same kinetic observed in untreated control macrophages (Fig. 5B). In recent years, additional inhibitory mechanisms that function later in the pathway and directly affect the active DNA-bound NF-kB have been identified. One of these mechanisms involves oxidation of redox-sensitive cysteine located in the DNA-binding loop; a cysteine residue in a well-conserved motif in all Rel/kB proteins is critical and must be maintained in a reduced state to allow DNA binding [38], [39]. Thus, it could be speculated that GSH-C4, by increasing the intracellular GSH content, may control NF-kB DNA binding activity favouring and prolonging its association with IL-12 p40 promoter sequence.

**Figure 7:**
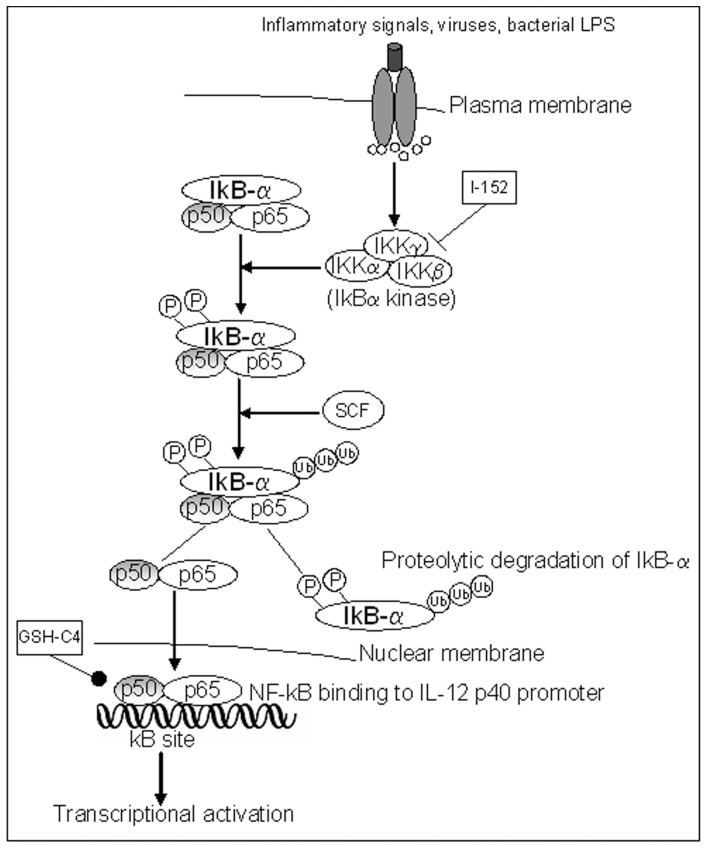
The canonical NF-kB signalling pathway. The binding of ligand to a receptor leads to the recruitment and activation of an IKK complex which phosphorylates IkB-α leading to degradation by the proteasome. NF-kB then translocates to the nucleus to activate target genes regulated by kB sites, such as that of IL-12 p40. I-152, reducing intracellular GSH, may inhibit the enzymatic activity of IKK complex by glutathionylation; as a consequence IkB-α degradation and NF-kB nuclear translocation are inhibited as well. GSH-C4, increasing intracellular GSH, may maintain in a reduced state the cysteine located in the DNA-binding loop so favouring and prolonging NF-kB DNA binding. SCF: SCF complex, Skp, Cullin, F-box containing complex: multi-protein E3 ubiquitin ligase complex.

Contrarily to GSH-C4, I-152 inhibited IkB-α degradation and as a consequence NF-kB nuclear translocation. There is increasing interest in new possible mechanisms in the regulation of NF-kB activation, and data already reported in literature show that multiple points of redox regulation exist, including S-glutathionylation of the IkB kinase IKK-β and the S-glutathionylation of IkB-α which has functional consequences on catalytic activity and protein–protein interactions [40], [41]. In agreement with these findings, it has been shown that chemicals able to decrease intracellular GSH content inhibit IkB-α degradation [41]–[43]. Thus, it could be hypothesized that I-152, by decreasing intracellular GSH, may induce IKK-β and/or IkB-α glutathionylation, resulting in the inhibition of IkB-α degradation and consequently NF-kB nuclear entry (Fig. 7). On the other hand, I-152 determines an increase in thiol content in the form of NAC, a molecule which is known to interfere with IkB degradation and NF-kB activation [44].

**Figure 8:**
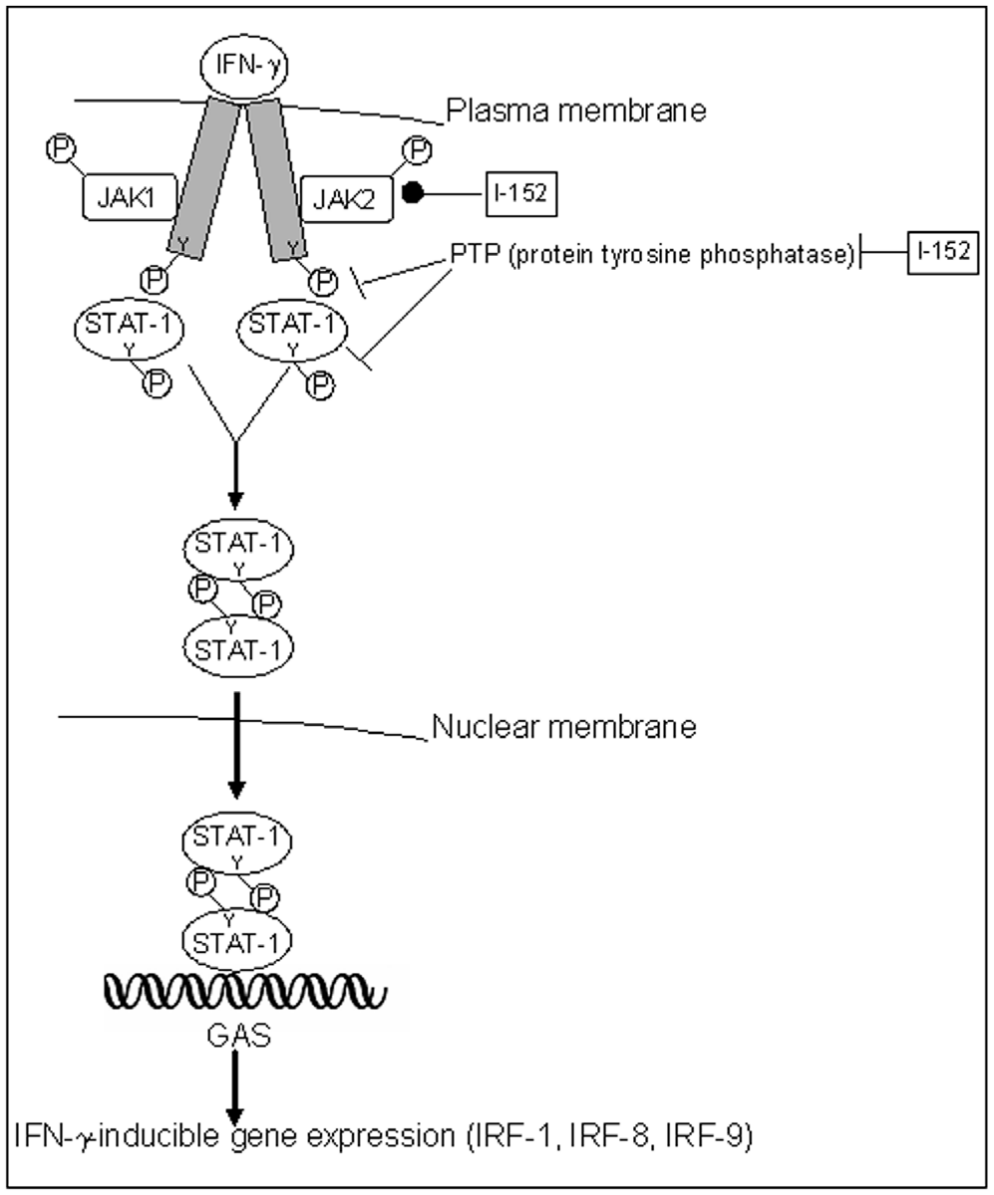
STAT-mediated activation of gene transcription by IFN-γ. The binding of ligand (IFN- γ) to the receptor results in receptor oligomerization and subsequent activation of receptor-associated JAK tyrosine kinases (JAK1 and JAK2). Activated JAKs phosphorylate specific tyrosine residues in the cytoplasmic domain of the receptor which in turn serves as the docking sites for the cytoplasmic transcription factors known as STAT-1. STAT-1 are therefore recruited to the phosphorylated receptor and subsequently phosphorylated by JAKs. The phosphorylated STAT-1 then dimerize, leave the receptor, and translocate to the nucleus where they activate the transcription of several genes, such as IRF-1. I-152, increasing intracellular thiol content, may positively influence JAK2’s catalytic activity which is known to be directly regulated by the redox state of the cell. I-152 may inhibit protein tyrosine phosphatases by glutathionylation. Y: tyrosine; GAS: IFN-γ activation site.

High concentrations of I-152 significantly increased IL-27 p28 production while GSH-C4 had no significant effects. The study of IRF-1 expression revealed that I-152 maintains the STAT-1/IRF-1 signalling pathway active for periods longer than controls. The persistence of STAT-1/IRF-1 signalling may explain the higher levels of IL-27 p28 found in macrophages treated with I-152. A possible mechanism by which I-152 could prolong activation of the STAT-1/IRF-1 signalling involves redox regulation of JAK2 through thiols provided by I-152 (in the form of NAC and I-152), since cysteine residues within JAK2 catalytic domain are crucial for enzymatic activity which is inhibited by oxidation and restored via reduction to the thiol state [45] (Fig. 8). Another mechanism may involve inhibition of tyrosine phosphatases since oxidation/reduction of conserved cysteinyl residues in the catalytic domains is an important factor in the regulation of their activity and a variety of redox-active metabolites can oxidatively inactivate protein tyrosine phosphatases with potentially profound implications for signal transduction [46]. Glutathionylation, as suggested above for inhibition of IkB degradation, can be proposed as possible mechanism for inhibition of tyrosine phosphatase by I-152. Having established that GSH-C4 and I-152 differentially modulate signalling pathway involved in IL-12 p40 and IL-27 p28 expression, further studies will be necessary to establish which step(s) of the NF-kB and STAT-1/IRF-1 signalling cascades may be selectively affected by GSH-C4 or I-152 treatment. It would also be interesting to study the contribution of GSH-C4 and I-152 to functional activity and expression of TLR and IFNGR as well as their influence on the intracellular molecules identified as essential for TLR-mediated signalling. In particular, by altering the intracellular thiol content, these molecules may influence the post-translational modification required for TLR function [47].

On the whole, these results suggest that it is possible to use different molecules to change the redox state of macrophages with the aim to induce Th1 cytokine production. In vivo we previously found that both GSH-C4 and I-152 increased circulating IL-12 levels and shifted the immune response towards Th1 in Ova- and Tat-immunized mice. The results reported here explain the possible mechanism through which GSH-C4 leads to an increase in IL-12 production while I-152 seems to have different effects in vivo and in vitro depending on the intracellular redox status. In fact, in animals immunized by an antigen, i.e. Ova, the content of GSH is reduced in APC because employed in the processing of antigens with disulfide bonds [1]–[3], [48], [49]. Thus, it could be hypothesized that I-152 is metabolized to replenish/increase GSH levels favoring NF-kB activation and IL-12 production. On the other hand, in vitro experiments here reported show that I-152 is able to enhance the production of the Th1 cytokine IL-27, favoring polarization towards Th1 profile. These results also suggest that increased IL-12 production is not the common effect of molecules that alter the redox state of APC but is the effect of GSH.

AIDS, cancer, allergic disorders, as well as aging, are characterized by a limited ability of organisms to mount a Th1 immune response against an infectious agent or a vaccine antigen. Of interest, GSH depletion in APC correlates with defective antigen processing and reduced secretion of Th1 cytokines, favoring polarization from the typical Th1 cytokine profile towards Th2 response patterns. In view of therapeutic interventions, one can envisage the application of GSH-C4 and I-152 both to interfere with viral infections, and to replenish intracellular GSH in APC favouring an efficient antiviral response and an efficient Th1 immune response.

## Materials and Methods

### Ethics Statement

Housing and treatment of mice were in compliance with the recommendations in the Guide for the Care and Use of Laboratory Animals by Health Ministry, law 116, 1992. Experiments were approved by the Committee on the Ethics of Animal Experiments of the University of Urbino “Carlo Bo”. The animals were suppressed by carbon dioxide. All efforts were made to minimize animal suffering and to reduce the number of animals used.

### Animals

Four-week-old female ICR(CD-1) mice were purchased from Harlan Nossan (Milan, Italy) and housed at 22±1°C with a 12 h light/dark cycle, 60±5% humidity, and 12 air changes/h. All animals were acclimatized for 1 week prior to use. Mice were.

### Reagents

I-152 was synthesized as previously described [20]. GSH-C4 was provided by Pepnome, Shanghai China.

### Murine Peritoneal Macrophage Isolation and Culture

The peritoneal exudate cells of ICR(CD-1) mice were obtained by peritoneal lavage with 10 ml of ice-cold Hank’s balanced salt solution supplemented with 10 U/ml of heparin. The cells were washed twice, resuspended in DMEM medium, supplemented with 10% fetal bovine serum (FBS) and penicillin–streptomycin, and overlaid (2×10^6^/plate) on plastic dishes (35 mm, Sarstedt, Italy). The plates were incubated in a humidified 5% CO_2_ atmosphere at 37°C overnight to allow macrophage adherence. Nonadherent cells were dislodged and the macrophages were incubated with either I-152 or GSH-C4 at different concentrations for 2 hours for thiol determination. The experiments in which IL-12 p40 and IL-27 p28 induction was studied, after 2 hours of incubation with the pro-GSH molecules, the cells were stimulated with 100 mg/ml LPS and 100 U/ml IFN-γ for different times according to the determination performed.

### High Performance Liquid Chromatography (HPLC) Determination of Thiols

Peritoneal macrophage cell cultures and RAW 264.7 cells (1×10^6^ cells/dish) were washed twice in warm Hank’s balanced salt solution, removed by scraping with a rubber policeman and immediately lysed with 100 µl of lysis buffer (0.1% Triton X-100, 0.1 M Na_2_HPO_4_, 5 mM EDTA, pH 7.5). Thereafter, 15 µl of 0.1 N HCl and 140 µl of precipitating solution (100 ml containing 1.67 g of glacial metaphosphoric acid, 0.2 g of disodium EDTA and 30 g of NaCl) were added. After centrifugation at 12,000×g for 10 min, the supernatants were collected and 25% (v/v) Na_2_HPO_4_ 0.3 M and immediately after 10% (v/v) DTNB were added. The mixture was stirred for 1 minute at room temperature (RT), then it was left at RT for another 5 minutes, and finally it was used for thiol determination by HPLC. A Teknokroma tracer excel 120 ODSA 5 µm 15×0.46 protected by a guard column was used in these studies. The mobile phase consisted of 10 mM KH_2_PO_4_ solution, pH 6.0 (buffer A) and buffer A containing 60% (v/v) of acetonitrile (buffer B). All buffer solutions, after preparation and pH adjustment, as well as standards, were filtered through 0.22-µm Millipore filter. The elution conditions were: 10 min 100% buffer A, 20 min up to 100% buffer B and hold 5 min. The gradient was returned to 100% buffer A in 3 min and the initial conditions restored in 4 min. The flow rate was 1 ml/min and detection was at 330 nm. Analyses were performed at room temperature and quantitative measurements were obtained by injection of standards of known concentration. The exact concentrations of standard solutions of GSH, GSH-C4 and I-152 were obtained by spectrophotometer readings at 412 nm following the procedure described by Beutler [36].

### Cytokine Assay

Total mouse IL-12 protein, both monomeric p40 and p40 associated to p35 (p70), and IL-27 p28 in the culture medium of peritoneal murine macrophages either treated or untreated with the pro-GSH molecules for 2 hours and then stimulated with 100 mg/ml LPS and 100 U/ml IFN-γ for 24 hours were determined by ELISA using kits from GE Healthcare UK Limited, Amersham Place, UK and R&D Systems. Inc. UK according to the manufacturer’s instructions. Standard curve for the cytokines was obtained using the recombinant standard protein provided by the manufacturer.

### Cell Toxicity Assay

Cytotoxicity was assessed by using a CellTiter-96 aqueous one solution kit from Promega (Madison, WI, USA). This assay is based on the reduction of the MTS reagent [3-(4,5-dimethylthiazol-2yl)-5-(3-carboxymethoxyphenyl)-2-(4-sulfophenyl)2H-tetrazolium, inner salt] into a colored formazan product that is soluble in tissue culture medium. This conversion is accomplished by NADPH or NADH produced by dehydrogenase enzymes in metabolically active cells. The quantity of formazan product, as measured by the absorbance at 490 nm, is directly proportional to the number of living cells in culture.

### IL-12 p40 and IL-27 p28 mRNA Quantification from Murine Peritoneal Macrophages

Interleukin mRNA levels were evaluated by quantitative-fluorescence-based real-time reverse transcription PCR (RT-PCR) in murine peritoneal macrophages incubated with either 20 mM GSH-C4 or 20 mM I-152 for 2 hours and then stimulated with 100 mg/ml LPS and 100 U/ml IFN-γ for 3 and 6 hours.

#### (i) RNA extraction and reverse transcription

Total RNA Purification Kit and RNase-Free DNase I Kit (Norgen, Biotek Corporation, Thorold, ON, Canada) were used for the isolation and purification of total RNA from macrophages and for maximum removal of residual genomic DNA respectively, according to the manufacturer’s instructions. Extracted RNAs were quantified with the NanoDrop ND-1000 Spectrophotometer as recommended by the manufacturer (NanoDrop Technologies, Wilmington, DE, USA). The absorbance ratio at 260/280 and 260/230 were measured to control the purity of RNA and since all samples had a ratio of about 2.0, they were accepted as pure RNA. The integrity of RNA was also confirmed by checking ribosomal RNA with electrophoresis on a 1% agarose gel. One µg of total RNA was used for cDNA synthesis using the M-MuLV Reverse Transcriptase KIT (Diatheva, Fano, Italia) in order to obtain an efficient reverse transcription reaction.

#### (ii) IL-12 and IL-27 mRNA quantification

Adequate strategies for primer design were applied to select the pair of primers spanning the exon-exon junctions, specific for the amplification of 202 bp of IL-12 p40 and 236 bp of IL-27 p28 mRNAs (GenBank accession no. NM_008352.2 and NM_145636.1 for IL-12 and IL-27 respectively) according to Oligo Primer analysis software (version 6.65; Cascade, CO, USA). The primer sequences were as follows: IL-12f (5′ primer, 5′- TCCCTGCAGGGTCCGATCCT-3′) and IL-12r (3′ primer, 5′-CCTGGCTCTGCGGGCATTTA-3′), IL-27f (5′ primer, 5′-GCACAGGCACCTCCGCTTTCA-3′) and IL-27r (3′ primer, 5′- GCAGCAGCAGGTCCCGAACAG-3′).

Real time PCR reactions were performed with a Hot-Rescue Real-Time PCR Kit-SG (2×) (Diatheva, Fano, Italy), in a final volume of 50 µl containing 2.5 and 5 µl of the cDNA mixture (corresponding to 50 and 100 ng of reverse transcribed total RNA, assuming a 100% efficiency of reverse transcription reaction), and 0,1 µM of each primer. The cycling conditions were 95°C for 10 min, followed by 40 cycles of 95°C for 15 s, 65°C for 20 s, 72°C for 35 s, or 95°C for 15 s and 68°C for 45 s for IL-12 and IL-27 respectively. Moreover, to check for the presence of inhibitors in the PCR reaction, an internal control, GAPDH housekeeping gene (GenBank accession no. NM_008907.1) was analyzed. The primer sequences were: PPIA 5′ primer, 5′-TGGCTGGATGGCAAGCATGT-3′ and PPIA 3′ primer, 5′-GAGCAGATGGGGTAGGGACGC-3′. The cycling conditions were 10 min at 95°C followed by 40 cycles of 15 sec at 95°C, 45 sec at 68°C and no difference in amplification was found (data not shown). All tests were conducted in triplicate.

At the completion of each run, melting curves for the amplicons were measured by raising the temperature by 1.0°C from 55 to 99°C while monitoring fluorescence. The specificity of the PCR amplification was checked by examining the melting curve for Tm, its symmetry, and the lack of non-specific peaks. Melting curves were checked routinely to confirm the quantification of the desired products. PCR amplification and detection were performed by using an ABI Prism 7700 Sequence Detection System (Applied Biosystems, Foster City, CA, USA). The cycle threshold (Ct) value was determined automatically with the SDS software version 1.9.1 (Applied Biosystems).

An external DNA standard curve was used to quantify IL-12 p40 and IL-27 p28 mRNA and a relative quantification approach was used to determine the changes in steady-state mRNA levels.

Briefly, ten-fold serial dilutions from 10^5^ to 10 copies of plasmid standard containing a specific DNA fragment of IL-12 (p40) or IL-27 (p28) were amplified in the same run with unknown samples. The standard curves were created automatically by the Applied Biosystems software based upon Ct values. A curve was accepted when the slope was between −3.40 and −3.32 (97–100% efficiency) and when the minimum value of the correlation coefficient (R^2^) was 0.98. mRNA levels were normalized to 100 ng of total RNA and expressed as percentage (%) vs untreated stimulated macrophages.

### Preparation of Whole-cell Lysates and Nuclear-cytoplasmic Subcellular Fractionation

For whole-cell extract preparation, macrophages were directly harvested in SDS buffer: 50 mM Tris-HCl, pH 7.8, 0.25 M sucrose, 2% (w/v) SDS, supplemented with a commercially available cocktail of protease (Roche Applied Science, Indianapolis, IN, USA) and phosphatase (1 mM NaF, 1 mM Na_3_VO_4_) inhibitors. Lysates were boiled for 5 min, then sonicated at 100 Watts for 20 sec. Cell debris was removed by brief centrifugation (10 min at 12,000×g). Protein content was determined by the Lowry assay [50].

Cytosolic and nuclear extracts were obtained by low salt/detergent cell lysis followed by high salt extraction of nuclei as previously described [51]. After treatment, cells were extensively washed with cold PBS and lysed with Buffer A [10 mM Hepes/KOH pH 7.9, 1.5 mM MgCl_2_, 10 mM KCl, 1 mM dithiothreitol (DTT), 0.2 mM EDTA, 0.1% Nonidet-P40, supplemented with a cocktail of protease (Roche Applied Science) and phosphatase inhibitors (1 mM NaF, 1 mM Na_3_VO_4_)]. The cell suspension was then chilled on ice for 10 min before centrifugation at 10,000×g. The supernatant, corresponding to the cytosolic fraction, was then transferred to a fresh tube, while the resultant pellet was suspended in Buffer B [20 mM Hepes/KOH pH 7.9, 25% glycerol, 0.42 M NaCl, 1.5 mM MgCl_2_, 1 mM DTT, 0.2 mM EDTA, supplemented with the cocktail of protease (Roche Applied Science) and phosphatase inhibitors] and incubated on ice for 20 min before being centrifuged at 10,000×g. Nuclear extract supernatant was collected, diluted 1∶4 in Buffer C (20 mM Hepes/KOH pH 7.9, 20% glycerol, 50 mM KCl, 1 mM DTT, 0.2 mM EDTA, 4 mM AEBSF) and stored in aliquots at –80°C until use.

### Western Immunoblotting

Protein extracts were resolved by SDS-PAGE and gels were electroblotted onto a nitrocellulose membrane (0.2 µm pore size) (BioRad laboratories Inc., Milano, Italy). The blots were probed with the primary antibodies listed below and bands were detected using horseradish peroxidase-conjugated secondary antibody (BioRad Laboratories Inc.). Peroxidase activity was detected with the enhanced chemiluminescence detection method (ECL Kit, Amersham Biosciences, Arlington Heights, IL, USA). The antibodies used in this study were: anti-IkB-α (C-21, sc-371), anti p65 (C-20, sc-372) and IRF-1 (M-20, sc-640) from Santa Cruz Biotechnology Inc. (Santa Cruz, CA, USA); anti-actin (A 2066) from Sigma-Aldrich; anti-Phospho-Stat1 (Tyr701) from Cell Signalling Technology (Beverly, MA, USA).

Densitometric quantitation of immunoreactive bands was performed in a Chemidoc apparatus (BioRad) equipped with the Quantity One software.

### Electrophoretic Mobility Shift Assay (EMSA)

Upper strand (5′-TCAACAGAGGGGACTTTCCGAGAGGCC-3′) and reverse-complement phosphodiester oligonucleotides, containing the NF-kB binding sequence (underlined), found in the enhancer of the immunoglobulin light chain gene (Igk) were custom synthesized by Thermo Fisher Scientific GmbH (Ulm, Germany) as HPLC-purified products. The Igk double-stranded ODN was 5′ end-labeled with [γ-^32^P]-ATP (Perkin Elmer Inc., Waltham, MA, USA) and T4 polynucleotide kinase (T4 PNK, Roche Applied Science). Nuclear extracts (5 µg) were preincubated with 3 µg of double-stranded non-specific DNA competitor poly(dI-dC) (Amersham Biosciences) for 10 min on ice in binding buffer (20 mM Hepes-KOH, pH 7.9, 0.1 M KCl, 5% (v/v) glycerol, 0.2 mM EGTA, 0.2 mM EDTA, 1 mM DTT). After this time, the labeled DNA probe was added to the mixtures at a final concentration of 4.4 nM and the incubation was continued for an additional 30 min. Reaction mixtures were then subjected to electrophoretic separation on 5% native polyacrylamide gels (29∶1 cross-linked) in Tris-glycine buffer (25 mM Tris base, 192 mM glycine). DNA/protein complexes were detected by exposing the dried gel in a Molecular Imager (BioRad Laboratories Inc.).

### Statistical Analysis

Statistical analysis of all data was performed with the Mann-Whitney test except for data of intracellular thiol content that were analysed by the Student’s t-test. P<0.05 was considered to be significant.

## References

[1] Short S, Merkel BJ, Caffrey R, McCoy KL (1996) Defective antigen processing correlates with a low level of intracellular glutathione. Eur J Immunol 26: 3015–3020. 8977298 10.1002/eji.1830261229

[2] Weiskopf D, Schwanninger A, Weinberger B, Almanzar G, Parson W, et al (2010) Oxidative stress can alter the antigenicity of immunodominant peptides. J Leuk Biol 87: 1–8. 10.1189/jlb.0209065 19801502

[3] Preynat-Seauve O, Coudurier S, Favier A, Marche PN, Villiers C (2003) Oxidative stress impairs intracellular events involved in antigen processing and presentation to T cells. Cell stress & Chaperones 8: 162–171. 14627202 10.1379/1466-1268(2003)008<0162:osiiei>2.0.co;2 PMC514868

[4] Koike Y, Hisada T, Utsugi M, Ishizuka T, Shimizu Y, et al (2007) Glutathione redox regulates airway hyperresponsiveness and airway inflammation in mice. Am J Respir Cell Mol Biol 37: 322–329. 17507665 10.1165/rcmb.2006-0423OC

[5] Murata Y, Shimamura T, Hamuro J (2002) The polarization of T(h)1 T(h)2 balance is dependent on the intracellular thiol redox status of macrophages due to the distinctive cytokine production. Int Immunol 14: 201–212. 11809739 10.1093/intimm/14.2.201

[6] Murata Y, Amao M, Yoneda J, Hamuro J (2001) Intracellular thiol redox status of macrophages directs the Th1 skewing in thioredoxin transgenic mice during aging. Molec Immunol 38: 747–757. 10.1016/s0161-5890(01)00111-0 11841834

[7] Dobashi K, Ahiara M, Araki T, Shimizu Y, Utsugi M, et al (2001) Regulation of LPS induced IL-12 production by IFN-γ and IL-4 through intracellular glutathione status in human alveolar macrophages. Clin Exp Immunol 124: 290–296. 11422207 10.1046/j.1365-2249.2001.01535.x PMC1906042

[8] Kamide Y, Utsugi M, Dobashi K, Ono A, Ishizuka T, et al (2011) Intracellular glutathione redox status in human dendritic cells regulates IL-27 production and T-cell polarization. Allergy 66: 1183–1192. 21545428 10.1111/j.1398-9995.2011.02611.x

[9] Murphy KM, Reiner SL (2002) The lineage decisions of helper T cells. Nat Rev Immunol 2: 933–944. 12461566 10.1038/nri954

[10] Trinchieri G (2003) Interleukin-12 and the regulation of innate resistance and adaptive immunity. Nat Rev Immunol 3: 133–146. 12563297 10.1038/nri1001

[11] Utsugi M, Dobashi K, Koga Y, Shimizu Y, Ishizuka T, et al (2002) Glutathione redox regulates lipopolysaccharide-induced IL-12 production through p38 mitogen-activated protein kinase activation in human monocytes: role of glutathione redox in IFN-γ priming of IL-12 production. J Leuk Biol 71: 339–347. 11818456

[12] Alam K, Ghousunnissa S, Nair S, Valluri VL, Mukhopadhyay S (2010) Glutathione-redox balance regulates c-rel-driven IL-12 production in macrophages: possible implications in antituberculosis immunotherapy. J Immunol 184: 339–347. 10.4049/jimmunol.0900439 20164428

[13] Aihara M, Dobashi K, Akiyama M, Naruse I, Nakazawa T, et al (2000) Effects of N-acetylcysteine and ambroxol on the production of IL-12 and IL-10 in human alveolar macrophages. Respiration 67: 662–671. 11124650 10.1159/000056297

[14] Pflanz S, Timans JC, Cheung J, Rosales R, Kanzler H, et al (2002) IL-27, a heterodimeric cytokine composed of EBI3 and p28 protein, induces proliferation of naïve CD(+) T cells. Immunity 16: 779–790. 12121660 10.1016/s1074-7613(02)00324-2

[15] Batten M, Ghilardi N (2007) The biology and therapeutic potential of interleukin 27. J Biol Med 85: 661–672. 10.1007/s00109-007-0164-7 17294231

[16] Fraternale A, Paoletti MF, Dominici S, Caputo A, Castaldello A, et al (2010) The increase in intra-macrophage thiols induced by new pro-GSH molecules directs the Th1 skewing in ovalbumin immunized mice. Vaccine 28: 7676–7682. 20875491 10.1016/j.vaccine.2010.09.033

[17] Fraternale A, Paoletti MF, Dominici S, Buondelmonte C, Caputo A, et al (2011) Modulation of Th1/Th2 immune responses to HIV-1 Tat by new pro-GSH molecules. Vaccine 29: 6823–6829. 21816192 10.1016/j.vaccine.2011.07.101

[18] Palamara AT, Brandi G, Rossi L, Millo E, Benatti U, et al (2004) New synthetic glutathione derivatives with increased antiviral activities. Antivir Chem Chemother 15: 83–91. 15185726 10.1177/095632020401500204

[19] Sgarbanti R, Nencioni L, Amatore D, Coluccio P, Fraternale A, et al (2011) Redox regulation of the influenza hemagglutinin maturation process: a new cell-mediated strategy for anti-influenza therapy. Antioxid Redox Signal 15: 593–606. 21366409 10.1089/ars.2010.3512

[20] Oiry J, Mialocq P, Puy JY, Fretier P, Dereuddre-Bosquet N, et al (2004) Synthesis and biological evaluation in human monocyte-derived macrophages of N-(N-acetyl-l-cysteinyl)-S-acetylcysteamine analogues with potent antioxidant and anti-HIV activities. J Med Chem 47: 1789–1795. 15027871 10.1021/jm030374d

[21] Fraternale A, Paoletti MF, Casabianca A, Orlandi C, Schiavano GF, et al (2008) Inhibition of murine AIDS by pro-glutathione (GSH) molecules. Antiviral Res 77: 120–127. 18164447 10.1016/j.antiviral.2007.11.004

[22] Gubler U, Chua A, Schoenhaut D, Dwyer C, McComas W, et al (1991) Co-expression of two distinct genes is required to generate secreted bioactive cytotoxic lymphocyte maturation factor. Proc. Natl. Acad. Sci. USA 88: 4143–4147. 10.1073/pnas.88.10.4143 PMC51614 1674604

[23] Takeda A, Hamnao S, Yamanaka A, Hanada T, Ishibashi T, et al (2003) Cutting edge: role of IL-27/WSX-1 signalling for induction of T-bet through activation of STAT1 during initial Th1 commitment. J Immunol 170: 4886–4890. 12734330 10.4049/jimmunol.170.10.4886

[24] Artis D, Johnson LM, Joyce K, Saris C, Villarino A, et al (2004) Cutting edge: early IL-4 production governs the requirement for IL-27-WSX-1 signalling in the development of protective Th1 cytokine responses following Leishmania major infection. Immunol 172: 4672–4675. 10.4049/jimmunol.172.8.4672 15067040

[25] Murphy TL, Cleveland MG, Kulesza P, Magram J, Murphy KM (1995) Regulation of interleukin 12 p40 expression through an NF-kB half-site. Mol Cell Biol 17: 5258–5267. 10.1128/mcb.15.10.5258 PMC230773 7565674

[26] Beg AA, Baldwin AS (1993) The I kappa B proteins: Multifunctional regulators of Rel/NF-kappa transcription factors. Genes Dev 7: 2064–2070. 8224838 10.1101/gad.7.11.2064

[27] Ghosh S, May MJ, Kopp EB (1998) NF-kB and Rel proteins: Evolutionary conserved mediators of immune response. Annu Rev Immunol 16: 225–260. 9597130 10.1146/annurev.immunol.16.1.225

[28] Hayden MS, Ghosh S (2004) Signalling to NF-kB. Genes Dev 18: 2195–2224. 15371334 10.1101/gad.1228704

[29] Liu J, Guan X, Ma X (2007) Regulation of IL-27 p28 gene expression in macrophages through MyD88- and interferon-γ-mediated pathways. J Exp Med 204: 141–152. 17227910 10.1084/jem.20061440 PMC2118415

[30] Zhang J, Qian X, Ning H, Yang J, Xiong H, et al (2010) Activation of IL-27 p28 gene transcription by interferon regulatory factor 8 in cooperation with interferon regulatory factor 1. J Biol Chem 285: 21269–21281. 20435892 10.1074/jbc.M110.100818 PMC2898397

[31] Chatterjee-Kishore M, Kishore R, Hicklin DJ, Marincola FM, Ferrone S (1998) Different requirements for signal transducer and activator of transcription 1α and interferon regulatory factor 1 in the regulation of low molecular mass polypeptide 2 and transporter associated with antigen processing 1 gene expression. J Biol Chem 273: 16177–16183. 9632673 10.1074/jbc.273.26.16177

[32] Harada H, Takahashi E-I, Itoh S, Harada K, Hori T-A, et al (1994) Structure and regulation of the human interferon regulatory factor 1 (IRF-1) and IRF-2 genes: implications for a gene network in the interferon system. Mol Cell Biol 14: 1500–1509. 7507207 10.1128/mcb.14.2.1500-1509.1994 PMC358505

[33] Ihle JN, Witthuhn BA, Quelle FW, Yamamoto K, Thierfelder WE, et al (1994) Signalling by the cytokine receptor superfamily: JAKs and STATs. Trends Biochem Sci 19: 222–227. 8048164 10.1016/0968-0004(94)90026-4

[34] Oiry J, Mialocq P, Puy JY, Fretier P, Clayette P, et al (2001) NAC/MEA coniugate: a new potent antioxidant which increases the GSH level in various cell lines. Bioorg Med Chem Lett 11: 1189–1191. 11354374 10.1016/s0960-894x(01)00171-8

[35] Tietze F (1969) Enzymatic method for quantitative determination of nanogram amounts of total and oxidized glutathione: applications to mammalian blood and other tissues. Anal Biochem 27: 502–522. 4388022 10.1016/0003-2697(69)90064-5

[36] Beutler E (1984) Reduced glutathione (GSH). In: Red cell metabolism. A manual of biochemical methods. New York, USA Press. 131–133.

[37] Sen CK, Packer L (1996) Antioxidant and redox regulation of gene transcription. The FASEB J 10: 709–718. 8635688 10.1096/fasebj.10.7.8635688

[38] Hayashi T, Ueno Y, Okamoto T (1993) Oxidoreductive regulation of nuclear factor *k* B. Involvement of a cellular reducing catalyst thioredoxin. J Biol Chem 268: 11380–11388. 8496188

[39] Toledano MB, Ghosh D, Trinh F, Leonard WJ (1993) N-terminal DNA-binding domains contribute to differential DNA binding specificities of NFkB p50 and p65. Mol Cell Biol 13: 852–860. 8423807 10.1128/mcb.13.2.852-860.1993 PMC358968

[40] Reynaert NL, van der Vliet A, Guala AS, McGovern T, Hristova M, et al (2006) Dynamic redox control of NF-kappaB through glutaredoxin-regulated S-glutathionylation of inhibitory kappaB kinase beta. Proc Natl Acad Sci USA 103: 13086–13091. 16916935 10.1073/pnas.0603290103 PMC1559757

[41] Kil IS, Kim SY, Park J-W (2008) Glutathionylation regulates IkB. Biochem Biophys Res Comm 373: 169–173. 18555796 10.1016/j.bbrc.2008.06.007

[42] Seidel P, Roth M, Ge Q, Merfort I, S’ng CT, et al (2011) IkBα glutathionylation and reduced histone H3 phosphorylation inhibit eotaxin and RANTES. Eur Respir J 38: 1444–1452. 21719482 10.1183/09031936.00129610

[43] Lou H, Kaplowitz N (2007) Glutathione depletion down-regulates tumor necrosis factor (TNF) α-induced NF-kB activity via IkB kinase (IKK)-dependent and –independent mechanisms. J Biol Chem 282: 29470–29481. 17690092 10.1074/jbc.M706145200

[44] Li YQ, Zhang ZX, Xu YJ, Ni W, Chen SX, et al (2006) N-acetyl-L-cysteine and pyrrolidine dithiocarbamate inhibited nuclear factor-kappa B activation in alveolar macrophages by different mechanisms. Acta Pharmacol Sin 27: 339–346. 16490171 10.1111/j.1745-7254.2006.00264.x

[45] Smith JK, Patil CN, Patlolla S, Gunter BW, Booz GW, et al (2012) Identification of a redox-sensitive switch within the JAK2 catalytic domain. Free Radic Biol Med 52: 1101–1110. 22281400 10.1016/j.freeradbiomed.2011.12.025 PMC3319112

[46] Salsman SJ, Hensley K, Floyd RA (2005) Sensitivity of protein tyrosine phosphatase activity to the redox environment, cytochrome c, and microperoxidase. Antioxid Redox Signal 7: 1078–1088. 15998263 10.1089/ars.2005.7.1078

[47] Dunston CR, Griffiths HR (2010) The effect of ageing on macrophage Toll-like receptor-mediated responses in the fight against pathogens. Clin Exp Immunol 161: 407–416. 20646005 10.1111/j.1365-2249.2010.04213.x PMC2962957

[48] Frosch S, Bonifas U, Eck HP, Bockstette M, Droege W, et al (1993) The efficient bovine insulin presentation capacity of bone marrow-derived macrophages activated by granulocyte-macrophage colony-stimulating factor correlates with a high level intracellular reducing thiols. Eur J Immunol 23: 1430–1434. 8325319 10.1002/eji.1830230704

[49] Ichiki H, Hoshino T, Kinoshita T, Imaoka H, Kato S, et al (2005) Thioredoxin suppresses airway hyperresponsiveness and airway inflammation in ashma. Biochem Biophys Res Commun 334: 1141–1148. 16039995 10.1016/j.bbrc.2005.07.007

[50] Lowry OH, Rosebrough NJ, Farr AL, Randall RJ (1951) Protein measurement with the Folin phenol reagent. J Biol Chem 193: 265–275. 14907713

[51] Crinelli R, Carloni E, Giacomini E, Penna A, Dominici S, et al (2012) Palytoxin and an Ostreopsis toxin extract increase the levels of mRNAs encoding inflammation-related proteins in human macrophages via p38 MAPK and NF-κB. PloS One 7: e38139. 22675515 10.1371/journal.pone.0038139 PMC3365899

